# Clinical characteristics and outcome of *Penicillium marneffei* infection among HIV-infected patients in northern Vietnam

**DOI:** 10.1186/1742-6405-9-24

**Published:** 2012-08-16

**Authors:** Mattias Larsson, Lien Ha Thi Nguyen, Heiman FL Wertheim, Trinh Tuyet Dao, Walter Taylor, Peter Horby, Trung Vu Nguyen, Minh Ha Thi Nguyen, Thuy Le, Kinh Van Nguyen

**Affiliations:** 1 Oxford University Clinical Research Unit, Welcome Trust Major Overseas Program, Hanoi, Vietnam; 2 Centre for Tropical Medicine, Nuffield Department of Clinical Medicine, University of Oxford, Oxford, UK; 3 National Hospital for Tropical Diseases, Hanoi, Vietnam; 4 Hawaii Center for AIDS, University of Hawaii at Manoa, John A. Burns School of Medicine, Honolulu, HI, USA

**Keywords:** Penicillium Marneffei, Penicillosis, HIV, Opportunistic Infections

## Abstract

**Objective:**

This study reports the clinical characteristics and outcome of HIV-associated *Penicilliummarneffei* infection in northern Vietnam.

**Methods:**

We conducted a retrospective chart review of all patients with laboratory confirmed *Penicilliummarneffei* infection admitted to the National Hospital for Tropical Diseases in Hanoi, Vietnam, between July 2006 and September 2009.

**Results:**

127 patients with *P. marneffei* infection were identified. All were HIV-infected; median CD4+ T-cell count was 24 cells/μl (IQR:12-48); 76% were men. Common clinical features were fever (92.9%), skin lesions (82.6%), hepatomegaly (61.4%), lymphadenopathy (40.2%), weight loss (59.1%) and cough (49.6%). Concurrent opportunistic infections were present in 22.0%; half of those had tuberculosis. Initial treatment regimens were: itraconazole or ketoconazole capsule (77.2%), amphotericin B (20.5%), and fluconazole (1.6%). In-hospital mortality was 12.6% and showed no significant difference in patients treated with itraconazole (or ketoconazole) and amphotericin B (p = 0.43). Dyspnea, ascites, and increased LDH level were independent predictors of mortality. No seasonality was observed.

**Conclusion:**

The clinical features, treatments and outcomes of HIV-associated *P. marneffei* infection in northern Vietnam are similar to those reported in other endemic regions. Dyspnea was an important predictor of mortality. More patients were treated with itraconazole than amphotericin B and no significant difference in treatment outcome was observed. It would be of clinical value to compare the efficacy of oral itraconazole and amphotericin B in a clinical trial.

## Background

*Penicillium marneffei* can cause a fatal systemic mycosis in immunosuppressed patients and is one of theleading causesof mortality in people living with Human Immunodeficiency Virus (HIV) in South-East Asia [1-3]. Penicilliosis presents primarily as a disseminated disease in HIV-infected patients with CD4+ T-cell count <100 cells/μL, involving the blood stream, skin, liver, spleen, lymph nodes, bone marrow, lung and gastrointestinal tract [2,4]. Typical umbilicated skin lesions are present in 70% of patients, facilitating early empirical antifungal therapy and resulting in better outcomes [4]. Laboratory diagnosis is made by microscopy and culture of skin lesions, blood, lymph node, or other body fluids [3].The majority of patients respond well to either amphotericin B or itraconazole treatment [2,4-6]; however no randomized controlled trials have been conducted to evaluate treatment choices for penicilliosis.

### Study design

We conducted a retrospective patient chart review of all patients with a compatible clinical syndrome and culture confirmed *P. marneffei* infection admitted to the National Hospital for Tropical Diseases (NHTD) in Hanoi between July 2006 and September 2009. Data collected included: demographics, admission date, clinical characteristics, HIV status, CD4+ T-cell count, laboratory investigations, concurrent opportunistic infections (OIs), treatment, and hospital outcome. *P. marneffei* was cultured from clinical specimens according to standard culture techniques [7]. For seasonality analysis, we assessed the number of penicilliosis admissions during dry versus rainy months in relation to all HIV-related admissions identified from hospital records. For outcome analysis three variables were used: 1. Survival including clinical improvement, defined as regression of symptoms such as fever, skin lesions, lymphadenopathy, hepatomegaly and splenomegaly, or resolution; 2. death at hospital discharge; 3. Lost to follow up due to that the patient were taken home or referred to another health facility and could not be contacted. We performed univariate and multivariate logistic regressions using forward stepwise selection of predictor variables. Statistically significant results (p < 0.05) from the univariate analysis were entered into a multivariate logistic regression model where continuous variables were dichotomized based on the mean values and after exclusion of correlated variables. The analysis was performed using Statistics Package of Social Sciences (SPSS version 19, USA).

## Results

*P. marneffei* infection was diagnosed in 127 patients, 42.5% by both blood and skin lesion culture, 29.1% by blood culture alone, and 28.3% by skin lesion culture alone. The average age was 32 years (range 21–50); the majority was male (75.6%), and 81.9% came from provinces outside of Hanoi in northern Vietnam. The reported route of HIV infection was intravenous drug use (IDU) (37.0%), commercial sex (29.9%), husband to wife (15.7%), combination of IDU and commercial sex (7.9%) and unknown (9.0%). 29.9% had been on antiretroviral therapy (ART) prior to the diagnosis of penicilliosis; the mean duration of ART was 10.8 weeks (SD 18.4).

The clinical and laboratory features are shown in Table 1 and Table 2. The median CD4+ T-cell count on admission was 24 cells/μl (IQR: 12–48). Other OIs were diagnosed (n = 28, 22%) patients; including tuberculosis(n = 14, 11.0%), *Pneumocystis jirovecii* pneumonia (PCP) (n = 6, 4.7%), varicella-zoster virus (n = 4, 3.1%) as well as toxoplasmosis, cytomegalovirus retinitis and herpes simplex virus type 1 infection all (n = 1, 0.8%). Five patients also had bacteremia with the following pathogens: *Escherichia coli*, *Staphylococcus aureus*, *Streptococcus pyogenes* and *Salmonella spp*. Six patients were previously diagnosed and received treatment for penicilliosis; however, none had started ART. In addition to blood and skin lesion cultures, there were 9 CSF cultures performed of which one was positive for *P. marneffei* and 11 lymph node aspirate cultures were all negative.

**Table 1:** Clinical features and association with outcome in 127 patients with penicilliosis

**Characteristics**	**All N = 127 (100%)**	**Fatal Cases N = 16 (13%)**	**Non Fatal Cases N = 111 (87%)**	**P value**	**Odds Ratio**	**95% Confidence Interval**
	**N (%)**	**N (%)**	**N (%)**			
Male sex	96 (76%)	12 (75%)	84 (76%)	NS		
Age	Mean 32 (SD 5.5)	Mean 32 (SD 5.7)	Mean 32 (SD 5.3)	NS		
**Symptoms/Signs**	N (%)	N (%)	N (%)			
Fever	118 (93%)	16 (100%)	102 (92%)	0.285	1.2	1.1 - 1.2
Skin lesions	105 (83%)	13 (81%)	92 (83%)	0.552	0.9	0.2 – 3.4
Weight loss	75 (59%)	8 (50%)	67 (60%)	0.300	0.7	0.2 – 1.9
Cough	63 (50%)	6 (38%)	57 (51%)	0.222	0.6	0.2 – 1.7
Diarrhoea	34 (27%)	6 (38%)	28 (25%)	0.233	1.8	0.6 – 5.3
Lymphadenopathy	51 (40%)	5 (31%)	46 (41%)	0.310	0.6	0.2 – 2.0
Hepatomegaly	78 (61%)	12 (75%)	66 (59%)	0.180	2.0	0.6 – 6.7
***Splenomegaly***	**46 (37%)**	**10 (62%)**	**37 (33%)**	**0.025**	**3.3**	**1.1 – 9.9**
***Ascites***	**29 (23%)**	**7 (44%)**	**22 (20%)**	0.040 0.048*	3.1 4.42*	1.1 – 9.4 1.01-19.32*
***Jaundice***	**17 (15%)**	**5 (31%)**	**12 (11%)**	**0.041**	**3.8**	**1.1 – 12.6**
***Dyspnoea***	**11 (9%)**	**6 (38%)**	**5 (4%)**	0.000 .013*	12.7 9.50*	3.3 – 49.2 1.62-55.68*

* Multiple regressionanalysis; SD. standard deviation.

**Table 2:** Laboratory test results of 127 patients with penicilliosis

**Laboratory findings**	**All N = 127 (100%)**	**Fatal Cases N = 16 (13%)**	**Non Fatal Cases N = 111 (87%)**		**P value**
	**Mean (SD) N**	**Mean (SD) N**	**Mean (SD) N**		
Hemoglobin (g/L)	92 (26) 126	95 (24) 16	92 (26) 110		0.666
***AST***(U/L)	**189 (183) 125**	**285 (238) 16**	**175 (170) 109**		**0.024**
ALT(U/L)	82 (81) 125	78 (71) 16	82 (83) 109		0.841
Albumin (g/L)	33.9 (8.4) 101	30.2 (8.3) 16	34.6 (8.3) 85		0.055
Total bilirubin (μmol/L)	37.5 (54.4) 85	49 (57) 16	35 (54) 109		.410
***LDH (***U/L)	**1104 (1378) 92**	**2501 (2662) 15**	**832 (714) 77**		**0.000**
					0.049*
CD4 + T-cell count (cells/μL)	36.9 (37.6) 114	31 (32) 10	38 (38) 104		0.401
***White blood cell count***(cells/μL)	**5777 (4063) 105**	**8667 (654) 9**	**5506 (369) 96**		**0.025**
***Platelets*** (cells/μL)	**134 (100) 126**	**91 (81) 16**	**141 (101) 110**		**0.037**
***Prothrombin time***	**74.4 (28.1) 54**	**42 (19) 10**	**82 (25) 44**		**0.000**
Creatinine (μmol/L)	95.2 (69.4) 120	103 (41) 16	94 (73) 104		0.477
***Urea*(**mmol/L)	**5.96 (3.32) 123**	**8.2 (4.0) 16**	**5.6 (3.1) 107**		**0.003**
	**Fatal Cases N = 16 (13%)**	**Non Fatal Cases N = 111 (87%)**	**P value**	**Odds Ratio**	**95% Confidence Interval**
***Positive blood culture***	**16 (100%)**	**75 (68%)**	**.003**	**1.2**	**1.1 – 1.3**
Positive skin culture	8 (50%)	82 (74%)	0.075	0.4	0.1 – 1.0

* Significant in binary multiple regression where LDH was dichotomized based on the mean value 1104 with 22 above and 70 below (OR 3.81 95% CI: 1.0-14.4), SD, standard deviation.

The initial choices of antifungal treatments are listed in Table 3. Outcome at discharge was clinical improvement or resolution in 107 (84.2%) patients and death in 16 (12.6%). Nine patients were discharged early due to request from the family members, or referred to other hospital. Five of them were assessed within a month after discharge and four patients were lost to follow-up. The mean duration of treatment in the hospital was 15.5 days (SD 11). For diseased patients deaths occurred early in the course of treatment after an average of 6.6 days (SD 10), compared to survivors, who were treated in average 16.7 days (SD 10)(p < 0.001). Among 98 patients receiving itraconazole or ketoconazole, 85 (86.7%) had an improvement; 13 (13.3%) died. By comparison, among 26 patients receiving amphotericin B, 24 (92.3%) had an improvement; and two (7.7%) patients died. The mortality differences between the two treatment groups did not reach statistical significance (p = 0.43, χ^2-^test). 

**Table 3:** Initial antifungal treatment and association with outcomes among126* patients with penicilliosis

	**All**	**Fatal Cases**	**Non Fatal Cases**	**Pvalue**	**Odds Ratio**	**95% Confidence Interval**
	**N = 126**	**N = 16**	**N = 110**			
	**(100%)**	**(13%)**	**(87%)**			
	**N (%)**	**N (%)**	**N (%)**			
Itraconazole	77 (61%)	11 (69%)	66 (60%)	0.222	1.8	0.6 – 5.5
Ketoconazole	4 (3%)	1 (6%)	3 (3%)	0.420	2.4	0.2 – 24.6
Amphotericin B	26 (21%)	2 (13%)	24 (22%)	0.35	0.5	1.1 – 2.6
Fluconazole	2 (2%)	1 (6%)	1 (1%)	0.237	7.3	0.4 – 123.5
Itraconazole or Ketoconazole**	17 (13%)	1 (6%)	16 (15%)	0.330	0.4	0.05 – 3.2

*Treatment data lacking for one patient.

**Itraconazoleand ketoconazole were used interchangeably based on availability.

Results of the logistic regression are shown in Table 1 for clinical variables and Table 2 for laboratory variables. Univariate analysis showed significantly higher risk of death among patients with dyspnea, defined as a combination of subjective sensation of difficulty breathing and observed tachypnea, ascites, jaundice, splenomegaly (Table 1), increased levels of alanine transaminase (AST), bilirubin, lactate dehydrogenase (LDH), white blood cell count, blood urea, thrombocytopenia and prolonged prothromb in time (Table 2). However, in the multivariate analysis, only dyspnea, ascites and increased LDH levels remained independent predictors of mortality. Of the 11 patients that died 6 had dyspnea, none of them had tuberculosis or *Pneumocystis jiroveci.* However, two were diagnosed with sepsis, one *Escherichia coli* and one *Streptococcus pyogenes.* In total 23 patients had X-ray confirmed lesions of the lungs, 5 of these also had dyspnea (p = 0.015), 4 had pulmonary tuberculosis and 2 had *Pneumocystis jiroveci*, 4 died, of those 2 were diagnosed with tuberculosis. Of the 5 patients with dyspnea and lung lesions one was diagnosed with *Pneumocystis jiroveci* and none with tuberculosis.

Assessment of seasonality was performed for the year 2007 and 2008. During these two years *P.marneffei* accounted for 87 of 793 (11.0%) of all HIV related admissions. The number of penicilliosis admissions was 43 during the hot rainy months (May to October) and 44 during the cooler dry months (November to April). The number of all HIV-related admissions was 463 during the rainy months and 330 during the dry months. The proportion of penicilliosis admissions in relation to all HIV-related admissions comparing dry versus rainy season did not show statistically significant difference (p = 0.07, χ^2-^test).

## Discussion

Penicilliosis accounted for 11% of all HIV-related admissions at NHTD in Hanoi during 2007 and 2008 which is higher than the 4.4% reported from the major referral hospital for infectious diseases in Ho Chi Minh City, southern Vietnam [4]. However as NHTD is a specialized tertiary hospital, and most (82%) of the cases were referred, and because other epidemiological data were lacking, it cannot be concluded that there is a difference in disease prevalence between northern and southern Vietnam. The clinical features of disseminated penicillosis are consistent with other studies including profound immunosuppression (median CD4+ T-cell count: 24 cells/μl) and high rate of co-infections with other opportunistic pathogens [2,4,6]. One third of the patients were already on ART for in average 10 days, this indicates that many patients had an ongoing *P. marneffei* infection that was not revealed before initiation of ART, but was probably unmasked by immune reconstitution inflammatory syndrome (IRIS) after initiation of ART, this has earlier been reported in a few case studies [8,9].

In our study the presence of dyspnea, ascites, and high LDH levels on admission independently predicted hospital mortality. Of the six patients that had dyspnea and died no one had tuberculosis or *Pneumocystis jiroveci diagnosis,* however two had septicemia. This may indicate that pulmonary involvement (i.e. *P. marneffei* pneumonia) drives disease severity or that the disease severity result in pulmonary lesions. This is consistent with a prior study showing that high respiratory rates and dyspnea among penicilliosis patients predicted poor hospital outcome [4]. Of the 11 patients with dyspnea 5 also had lung lesions, of these one was diagnosed with *Pneumocystis jiroveci* and non with tuberculosis. Lung involvement of *P. marneffei* has been shown in Taiwan where it was found to be the most common cause of cavities in the lungs of immunosuppressed HIV infected patients [10]. The majority of patients with dyspnea did not have lung lesions, hence the dyspnea might be due to the severe condition with multi-organ involvement and acute respiratory distress syndrome (ARDS). It should be noted that there could be some under diagnosis of tuberculosis due to the poor sensitivity for sputum microcopy and culture in immunosuppressed individuals as well as for Pneumocystis jiroveci as microscopy of sputum, obtained by nebulizer or bronchoscopy, is not routinely performed.

Typical skin lesions were present in 80% of patients in this study. The pathogenesis and prognosis of skin involvement is poorly understood. Although a previous study showed that presence of typical skin lesions facilitates early initiation of empirical antifungal treatment and results in better outcome [4], it is unclear whether skin involvement itself is a prognostic marker. In the absence of skin lesions, the differential diagnoses for an AIDS-associated febrile illness with reticuloendothelial system involvement are broad and include: *Mycobacterium tuberculosisMycobacterium Avium* Complex, histoplasmosis and cryptococcosis among others [11]. This poses a major challenge in diagnosis and treatment, especially in areas with poor access to blood culture and other diagnostic laboratory. Penicilliosis should be considered in all severely immunosuppressed HIV patients with one or more of the common presentations, skin lesions, lymphadenopathy, hepatomegaly, splenomegaly, ascites, jaundice and dyspnoea, who have been in *P. marneffei* endemic areas, and empirical antifungal treatment in very ill patients may be indicated.

In this study 22% of the patients had a concurrent OI; of whom half had tuberculosis. Hence, multiple OIs, particularly pulmonary tuberculosis, need to be considered. Tuberculosis and penicilliosis co-infection poses a major therapeutic dilemma in resource-poor settings as rifampicin is a potent P450 inducer and markedly reduces itraconazole concentrations [12] Amphotericin B is recommended for patients with concurrent tuberculosis treatment. An alternative tuberculosis drug rifabutin is not available in most penicilliosis endemic areas.

The WHO recommended treatment for severe penicilliosis, amphotericin B, was only given to 20.5% of patients. The majority (77.2%) was treated with either itraconazole or ketoconazole. Although itraconazole is a recommended alternative treatment for mild to moderate disease or when amphotericin B is unavailable [13], in Vietnam both mild and severe cases are commonly treated with oral itraconazole because amphotericin B is often not available or is too expensive. In our study there was no significant difference between itraconazole and amphotericin B in treatment outcome. So far no randomized, prospective treatment trials have been conducted to compare the efficacy of different antifungal treatment regimens for penicilliosis.

Compared to northern Thailand and southern Vietnam where cases peak in the rainy season [4], seasonality was not observed in our cohort. It should be noted that our small sample size and short time frame does not enable any conclusive results about seasonality. However, the cool and dry season in northern Vietnam is often damp with high humidity which might have an impact on the reservoirs of *P. marneffei.*

This study shows that penicilliosis in northern Vietnam presents with similar clinical characteristics as in other endemic areas, and that dyspnea is an important predictor of mortality. It is common practice to treat patients with oral itraconazole rather than amphotericin B, and no significant difference in treatment outcome was observed. It would be of clinical value to compare the efficacy of oral itraconazole and amphotericin B in a clinical trial to develop evidenced based guidelines.

## Abbreviations

HIV, Human Immunodeficiency Virus; NHTD, National Hospital for Tropical Diseases; IDU, Intravenous drug use.

## Competing interests

The author declares that they have no competing interests

## Authors’ contributions

NTLH - conception and design, acquisition of data, analysis and interpretation of data, has been involved in drafting the manuscript and have given final approval of the version to be published. ML - Analysis and interpretation of data, has been involved in drafting the manuscript and have given final approval of the version to be published. HFLW - conception and design, acquisition of data, interpretation of data, revising it critically for important intellectual content and have given final approval of the version to be published. DTT – Laboratory analysis and have given final approval of the version to be published. WT - conception and design, acquisition of data, revising it critically for important intellectual content and have given final approval of the version to be published. PH -conception and design, acquisition of data, interpretation of data, revising it critically for important intellectual content and have given final approval of the version to be published. NVT– Laboratory analysis and have given final approval of the version to be published. NTMH - Acquisition of data and have given final approval of the version to be published. TL - Revising it critically for important intellectual content and have given final approval of the version to be published. NVK - conception and design, acquisition of data and have given final approval of the version to be published. All authors read and approved the final manuscript.
